# Cerebrovascular haemodynamics in preeclamptic patients

**DOI:** 10.1186/cc14244

**Published:** 2015-03-16

**Authors:** E Shifman, S Floka

**Affiliations:** 1 The State Budgetary Healthcare Institution of Moscow Area 'Moscow's Regional Research Clinical Institute n.a. M.F. Vladimirsky', Moscow, Russia

## Introduction

The goal of the study was to analyse cerebral blood flow in pregnancy complicated by preeclampsia.

## Methods

This was a prospective study. I group: 45 patients, 17 to 38 years (mean age 27.5 ± 5.3 years) with verified diagnosis of severe preeclampsia; control group: 72 healthy women with normal pregnancy, third trimester, 19 to 34 years (mean age 24.5± 4.3 years). Exclusion criteria: potentially haemodynamically significant stenosis; congestive heart disease; arrhythmia; large changes in haemorheology; diabetes mellitus; and craniospinal trauma and syncope. Study of cerebral flow was improved by the method of transcranial dopplerography (TCD). All patients underwent duplex scan of extracranial portions of brachiocephalic arteries and transcranial duplex scan in the area of middle cerebral artery (MCA) (segment M1). During duplex scan of brachiocephalic arteries lumen, the presence of extravasal causes for basic blood flow disturbances was estimated. We determined lumen of large basilar arteries and quantitative features of blood flow in MCA. By the transtemporal approach in the MCA M1 segment, peak systolic flow velocity (Vps), maximal end-diastolic velocity (Ved), time-adjusted maximal velocity (TAMX), resistance index (RI), pulsative index (PI), and systolic/diastolic ratio (S/D) were determined. Significance of mean value differences were calculated using the STATISTICA 6.0 program with determination of Student's *t *criteria with normal spread in the group.

## Results

All haemodynamic values in the M1 segment of MCA in preeclamptic patients were decreased in comparison with the same values in healthy pregnant women with different significance: PI (mean 0.77 vs. 0.84, *P *< 0.01); RI (mean 0.52 vs. 0.54, *P *< 0.05); Vps (mean 90.22 vs. 104.74 cm/second, *P *< 0.001); Ved (mean 43.25 vs. 48.53 cm/second, *P *< 0.001); TAMX (mean 61.48 vs. 67.30 cm/second, *P *< 0.01); and S/D (mean 2.02 vs. 2.06, *P *< 0.05). Found pathophysiological changes of cerebral haemodynamics were consistent with a dopplerographic pattern of diminished perfusion and are typical for vascular segments, which are located proximally to the zone of abnormally high haemodynamic resistance: prestenotic arterial segments, episodes of arterial hypertension and distal vasoconstriction.

## Conclusion

With TCD we obtained a possibility to determine and estimate changes in cerebrovascular flow in pregnant patients with severe preeclampsia. This enhances diagnostic possibilities of some serious pregnancy complications, and gives us deep understanding of some components of pathogenesis and increased treatment efficacy.

